# Salivary levels of total huntingtin are elevated in Huntington’s disease patients

**DOI:** 10.1038/s41598-018-25095-3

**Published:** 2018-05-09

**Authors:** Jody Corey-Bloom, Ameera S. Haque, Sungmee Park, Ajay S. Nathan, Robert W. Baker, Elizabeth A. Thomas

**Affiliations:** 1 Department of Neurosciences, University of California, San Diego, CA USA; 2 Department of Neuroscience, The Scripps Research Institute, La Jolla, CA USA

## Abstract

Patients with Huntington’s disease (HD), an autosomal-dominant neurodegenerative disease, show substantial variability in age-of-onset, symptom severity and course of illness, warranting the need for biomarkers to anticipate and monitor these features. The HD gene encodes the disease protein huntingtin (Htt), a potentially useful biomarker for this disease. In the current study, we determined whether total Htt protein (normal plus mutant; “tHtt”) could be reliably measured in human saliva, a body fluid that is much more accessible compared to cerebral spinal fluid or even blood, and whether salivary levels of tHtt were clinically meaningful. We collected 146 saliva samples from manifest HD patients, early-premanifest individuals, late-premanifest patients, gene-negative family members and normal controls. We found that tHtt protein could be reliably and stably detected in human saliva and that tHtt levels were significantly increased in saliva from HD individuals compared to normal controls. Salivary tHtt showed no gender effects, nor were levels correlated with total protein levels in saliva. Salivary tHtt was significantly positively correlated with age, but not age-of-onset or CAG-repeat length. Importantly, salivary tHtt was significantly correlated with several clinical measures, indicating relevance to disease symptom onset and/or severity. Measurements of salivary tHtt offer significant promise as a relevant, non-invasive disease biomarker for HD, and its use could be implemented into clinical applications.

## Introduction

The mutation responsible for Huntington’s disease (HD) is an abnormal CAG repeat expansion in the *HTT* gene^1,2^. Despite HD being a single gene disorder, there is enormous variability in disease onset and severity, even in patients with the identical CAG repeat number^3–5^. For example, HD patients with identical CAG repeat lengths, particularly those between 40–44, can have age of onsets that differ by more than 20 years^5^. Symptom presentation and course of illness also varies among patients, necessitating the identification of biomarkers to predict and/or track such features. The *HTT* gene encodes the protein huntingtin (Htt), a large protein of 3,144 amino acids, which could serve as a relevant biomarker. Htt is involved in a wide range of cellular functions including gene transcription, vesicle transport and energy metabolism^6,7^. Htt is an essential protein and is required for normal embryogenesis, as knock-out mice for *Htt* die at embryonic day 8.5^8–10^. In addition to being widely expressed in different cell types within the brain, Htt is also ubiquitously expressed in peripheral tissues and throughout the body^1,6^. Pathogenesis in HD arises largely from the expression of mutant Htt (mHtt), which leads to the formation of toxic soluble protein oligomers as well as insoluble aggregates that contribute to the disruption of many intracellular pathways^11^. While insoluble mHtt aggregates in the central nervous system (CNS) are the primary pathological hallmark of HD, the formation of polyglutamine inclusions also occurs in non-CNS tissues^12^. Accordingly, patients with HD exhibit multiple peripheral changes, including skeletal muscle deficits, metabolic abnormalities and immune system dysfunction^13^, which likely arise due to the expression of mHtt at peripheral sites.

Measurements of Htt in the brains of patients are not possible, hence several approaches have turned to measuring both normal and mHtt in other biological fluids, including cerebral spinal fluid (CSF)^14,15^ and blood^16,17^ from human patients in order to assess the utility of Htt to serve as a biomarker. However, CSF collection requires a lumbar puncture, which is highly invasive, and CSF levels of Htt are known to be very low^14^. Measurements of Htt in blood have been successful^16,17^, however levels vary according to the blood cell type, large starting volumes (i.e. 50 ml) are often required and blood drawing itself is also an invasive technique. As an alternative, in this study, we explored whether Htt could be reliably measured in saliva from HD patients and normal individuals. Saliva is a composite of oral fluids secreted from many different glands, including the major and minor salivary glands, as well as the buccal, lingual, and palatal tissues^18^. Saliva also contains cellular components, including leukocytes and buccal cells. Like blood, saliva contains a wealth of hormones, proteins and nucleic acid molecules that reflect physiological status^18^. Importantly, several other neurodegenerative disease-related proteins have been identified in human saliva, including amyloid beta^19^, tau^20^, alpha-synuclein^21^ and DJ-1^22,23^. Salivary levels of these proteins are thought to represent useful biomarkers for Alzheimer’s and Parkinson’s diseases, respectively. In this study, we have measured Htt protein in human saliva samples and explored the potential of salivary Htt to serve as a peripheral biomarker for HD. We suggest that salivary levels of Htt might represent a useful biomarker to monitor the effects of Htt-lowering therapies, several of which are already in clinical trials (NCT02519036, NCT03342053, NCT03225846, NCT03225833).

## Results

### Htt protein is present in saliva

**Figure 1:**
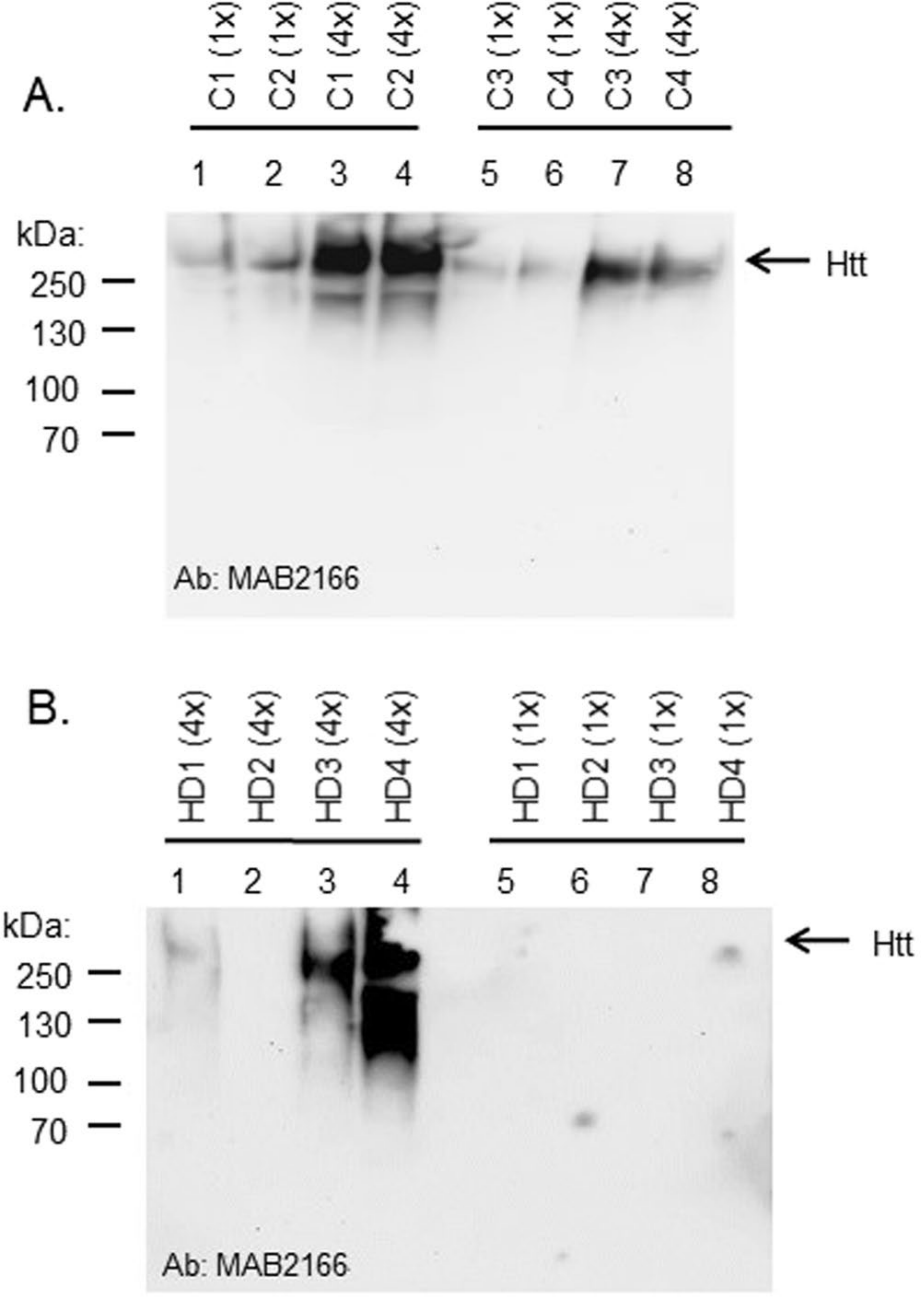
(**A**) Western blot of Htt protein in saliva from normal and HD individuals. Three milliliters of unstimulated whole saliva was collected from four normal individuals (C1-C4). Saliva supernatants were used as-is or were concentrated 4-fold by vacuum centrifugation. Supernatant fractions were separated by 7% SDS-PAGE and blotted onto nitrocellulose membranes for immunodetection using MAB2166 antibody, a monoclonal anti-huntingtin antibody which recognizes a fragment of the protein (amino acids 181–810), located outside the polyglutamine region. Gel image was acquired using a Fluorochem E imager. (**B**) Western blot of Htt protein in saliva from HD patients carried out as in (**A**).

To determine first whether the normal Htt protein was present in human saliva, we performed qualitative Western blotting using the MAB2166 anti-Htt antibody on eight samples from normal individuals. We detected a single band above the 250 kDa marker, corresponding to the full-length Htt protein (~350 kDa) in unconcentrated saliva samples (1x) (n = 4 in Fig. 1A, and n = 4 additional samples in Fig. S1A). In samples that had been concentrated four-fold, this signal increased approximately proportionally (Figs 1A, S1A). Similarly, in saliva samples from HD patients, we also detected Htt immunoreactivity corresponding to the full-length protein in 3 out of the 4 patient samples (Fig. 1B), however, levels were not readily detected in unconcentrated samples (Fig. 1B). Using a different anti-Htt antibody, 4E10, we also could detect Htt protein in HD patient samples, but not controls (Fig. S1B).

### Quantification of Htt by ELISA

**Figure 2:**
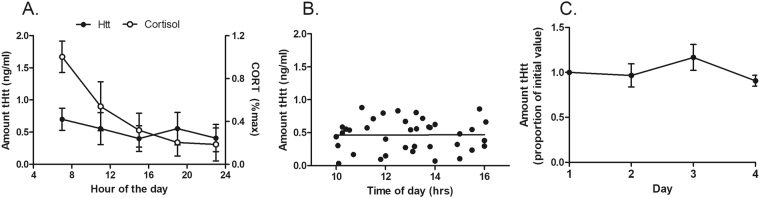
Diurnal stability and reproducibility of salivary tHtt levels. (**A**) Levels of tHtt were measured using ELISA in normal individuals over the course of the day and show no diurnal variation. For comparison, cortisol levels were measured using immunoassays from the same individuals at the same time points. The amount of tHtt reported is relative to a recombinant Htt protein standard corresponded to amino acids 802–940 of the human Htt protein. Data represent the mean +/− S.E.M value from n = 5 individuals. (**B**) Salivary levels of tHtt do not vary over the time of sample collection (10 am to 4 pm) in HD patients. Data represent the mean +/− S.E.M value from n = 40 patients. (**C**) Salivary tHtt is reproducibly detected on different days. Data shows tHtt measures taken on four non-consecutive days from the same individuals (n = 8).

We next used an ELISA method to quantify levels of Htt in saliva supernatants from normal individuals using antibodies that recognize total Htt protein levels (“tHtt”: normal plus mutant Htt protein). We first assessed whether the saliva matrix interfered with the ELISA assay by spiking-in a known amount of recombinant Htt protein to saliva samples from two normal individuals. We found that the recovery of Htt in the saliva matrix was 91.2% +/− 4.05% and that the levels of recombinant Htt diluted linearly in saliva (r^2^ = 0.976) (Fig. S2). We next determined whether salivary levels varied over the course of the day (7 am to 11 pm) in normal individuals, and the reliability of measurements taken on different days from the same individuals. Compared to salivary cortisol, which exhibits a known diurnal variation with peak levels observed upon wakening^24^, salivary tHtt in the same normal subjects did not vary over the course of the day (Fig. 2A). Consistent with this finding, salivary levels in a subset of n = 40 patients were not correlated with time of day of sampling (from 10 am to 4 pm) (Fig. 2B). Further, in patients who gave samples on four different days, there was remarkable consistency in the tHtt measurements taken on different days from the same person (Fig. 2C).

### Salivary tHtt distinguishes different diagnostic groups

**Figure 3:**
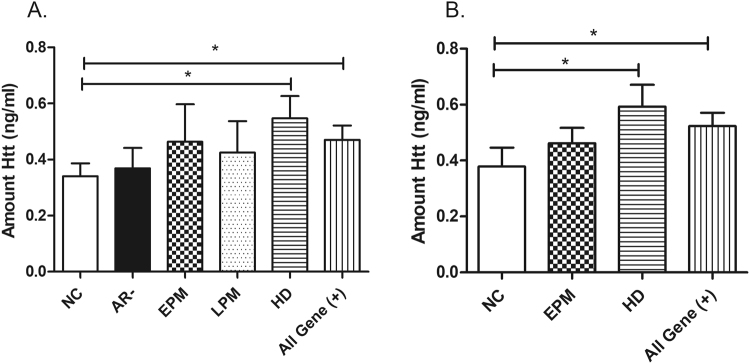
Levels of tHtt are elevated in saliva from HD patients. Total Htt levels (ng/ml) were measured using ELISA in saliva supernatants from five different diagnostic groups: NC = normal control; AR(−) = at-risk, gene-negative; EPM, Early premanifest; LPM, late pre-manifest; HD = manifest Huntington’s disease. The amount of tHtt (ng/ml) reported is relative to a recombinant Htt protein of 139 amino acids per the ELISA assay. Differences in salivary tHtt levels were determined by One-way ANOVA with a Dunnett’s post-test. *P < 0.05, **P < 0.01. An initial cohort, Cohort 1, and a validation cohort, Cohort 2, were used for this study (see Table 1).

We next determined whether tHtt levels in saliva were different according to different diagnostic groups comprising two cohorts of subjects, whose samples were collected approximately 8 months apart (see Table 1). There were no statistically significant differences in the mean ages among the different diagnostic groups. In cohort 1, we found that tHtt levels were significantly increased in saliva from manifest HD patients compared to normal controls (0.547 ± 0.079 vs. 0.340 ± 0.046 ng/ml; P = 0.022) (Fig. 3A). As expected, there was no significant difference in salivary tHtt levels between normal controls and gene-negative (At-risk negative [AR(−)]) subjects, who are offspring of an affected parent, but did not inherit the disease mutation (Fig. 3A). There was a trend towards higher levels of tHtt in saliva from early pre-manifest (EPM) patients compared to normal controls (0.464 ± 0.133 vs. 0.340 ± 0.046 ng/ml) (Fig. 3A), but the difference did not reach statistical significance. In a second cohort of patients, we observed similar results with EPM patients showing a higher mean level of tHtt compared to normal controls (0.462 ± 0.055 vs. 0.378 ± 0.068 ng/ml) (Fig. 3B), but this difference was not statistically significant. However, manifest HD patients showed statistically significantly higher levels of salivary tHtt compared to normal controls (0.593 ± 0.078 vs. 0.378 ± 0.068 ng.ml; P = 0.040) (Fig. 3B).

**Table 1:** Summary of subjects used in this study.

Cohort 1:					
	HD:	LPM:	EPM:	AR(−):	NC:
Number of patients	23	9	10	16	30
Female:Male	13:10	6:3	5:5	11:5	15:15
Avg age (yrs)	54.2	53.7	43.4	44.6	51.6
CAG repeat	43.2	42.2	41.2	23.1	NA
**Cohort 2:**					
	**HD:**	**EPM:**	**AR(−):**	**NC:**	
Number of patients	14	16	13	14	
Female:Male	9:5	9:7	6:7	8:6	
Avg age (yrs)	55	43.9	49.5	53	
CAG repeat	42.2	41.4	19.8	NA	

HD = Huntington’s disease; LPM = Late pre-manifest; EPM = Early pre-manifest; AR(−) = At Risk, gene negative; NC = Normal controls. NA = Not applicable. Subjects were comprised of two cohorts of subjects, whose samples were collected approximately 8 months apart. Approximately 20% of the subjects in cohort 2 also gave a sample for cohort 1 but contributed distinct clinical data at each time point.

**Figure 4:**
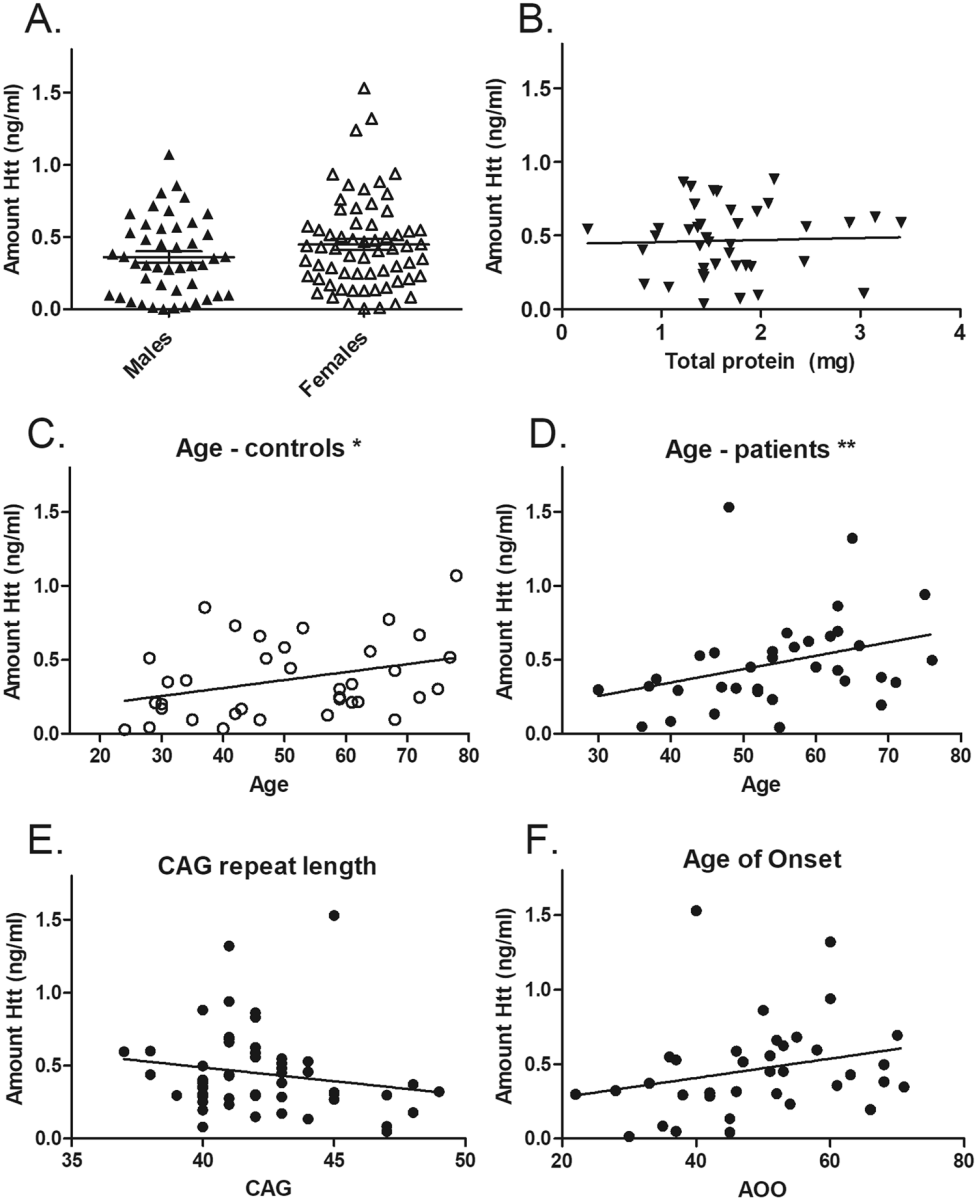
Salivary tHtt associations to sex, age, CAG repeat number and age-of-onset. (**A**) Salivary tHtt shows no significant differences between males and females (p = 0.135, Student’s t test; n = 117 subjects). (**B**) Levels of tHtt in saliva were not correlated with total protein in a subset of HD patients (n = 40 patients). A statistically significant correlation between salivary tHtt and age in (**C**) normal control (r = 0.375; p = 0.024) and (**D**). HD patients (r = 0.432; p = 0.0096) was observed. (**E**) Salivary tHtt levels were not statistically significantly correlated with CAG repeat length of all gene positive patients, however a trend towards a negative correlation was detected (r = −0.182; P = 0.189; n = 56). (**F**) No significant correlation was detected between salivary tHtt levels from manifest HD patients and age-of-onset (r = 0.203; p = 0.242; n = 36 patients).

We did not detect a significant effect of sex on salivary tHtt levels (Fig. 4A), nor was salivary tHtt associated with hydration status, as gauged by measuring total protein levels in each sample and correlating with tHtt amounts (Fig. 4B). We did observe statistically significant correlations between salivary tHtt and age in normal control (r = 0.375; p = 0.024) and HD patients (r = 0.432; p = 0.0096) (Fig. 4C and D, respectively). Salivary tHtt levels were not statistically significantly correlated with CAG repeat length of all gene positive patients, however a trend towards a negative correlation was detected (r = −0.182; P = 0.189) (Fig. 4E). No significant correlation was detected between salivary tHtt levels from manifest HD patients and age-of-onset (r = 0.203; p = 0.242) (Fig. 4F), although the observed positive trend is likely related to the age correlation observed in these patients (Fig. 4D).

### Correlations to clinical measures

**Figure 5:**
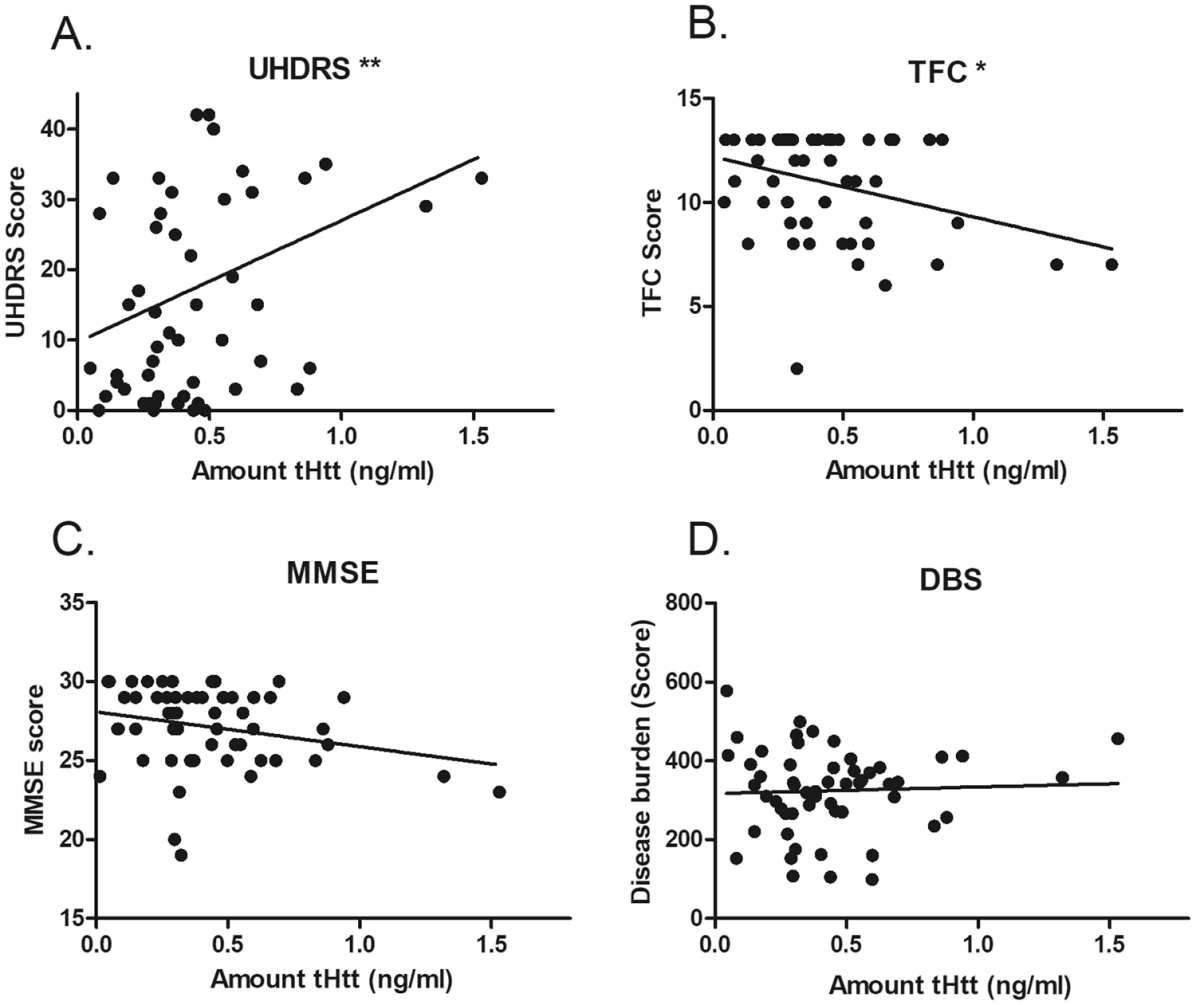
Salivary tHtt and correlations to clinical measures. Levels of tHtt in saliva from patients only (early pre-manifest (EPM), late pre-manifest (LPM) and HD) were used for analysis. Salivary tHtt was significantly positively correlated to the Unified Huntington’s Disease Rating Scale (UHDRS) score (**A**) (Spearman’s r = 0.364; p = 0.0068), and a significantly negatively correlated to the Total Functional Capacity (TFC) score (**B**) (Spearman’s r = −0.267; p = 0.049). No significant correlation was found to the Mental State Examination (MMSE) (**C**) or Disease Burden Score (DBS) (**D**).

We next assessed the severity of disease symptoms in EPM, LPM and manifest HD patients using various measures, including Mental State Examination (MMSE), the Unified Huntington’s Disease Rating Scale (UHDRS) and Total Functional Capacity (TFC) (Table S1) and determined whether these measures were correlated with tHtt levels in saliva samples from subjects in both cohorts 1 and 2. Salivary tHtt showed a statistically significant positive correlation to the UHDRS score (Spearman’s r = 0.364; p = 0.0068) and a significant negative correlation to the TFC score (Spearman’s r = −0.267; p = 0.049) (Fig. 5A,B). There was a trend towards a negative correlation between tHtt and MMSE (Spearman’s r = −0.252; p = 0.0632) (Fig. 5C). These findings suggest that salivary tHtt concentrations have clinical relevance, however no significant correlations were observed between tHtt disease burden scores (DBS) (Fig. 5D). With regards to medication status, about half of the HD patients were taking antidepressant medications of various types, including Paxil, Fluoxetine, Wellbutrin and Zoloft. Although the doses of these medications were not available, we did not observe differences in salivary tHtt between those patients taking any antidepressant medication compared to patients not taking medications (Fig. S3).

## Discussion

In HD, as well as other neurodegenerative diseases, there is an enormous need to identify easily accessible biomarkers which could be used to predict symptom onset, to assess symptom severity and/or to track potential therapeutic interventions. The disease protein, Htt, represents an ideal biomarker for such endeavors, provided that levels of Htt can be reliably measured in peripheral tissues. In this study, we demonstrate that tHtt protein can be detected in saliva and that salivary tHtt can be reproducibly measured using an ELISA assay. Importantly, we found that salivary tHtt was elevated in HD patients compared to normal controls and AR- family members and that salivary tHtt levels correlated with clinical measures of motor function.

Utilization of saliva as a biospecimen for biomarker research has gained attention only in recent years. Proteomic studies have found >2,000 proteins detected in saliva, 27% of which were also present in blood^25^. Moreover, nearly 40% of the proteins that have been suggested to be candidate markers for diseases such as cancer, cardiovascular disease, and stroke can be found in whole saliva^25^. More recently, with the detection of several neurodegenerative disease-related proteins in human saliva, including amyloid beta^19^, tau^20^, alpha-synuclein^21^ and DJ-1^22,23^, the utility of saliva to reveal information about brain diseases is especially exciting, although this field is in its infancy.

While tHtt is clearly present in saliva, the origin of salivary tHtt is unclear. Constituents from the blood can enter into the saliva via transcellular transport, passive intracellular diffusion or active transport; hence, salivary levels of tHtt could reflect the overall circulating levels in the body. If this is the case, then it is possible that the elevated levels of tHtt detected in HD patients could reflect leakage from the brain via a compromised blood-brain barrier, which has been reported in HD^26^. Studies comparing salivary levels with blood levels will be necessary to test this idea and these studies are currently underway. Cellular components of saliva include leukocytes and buccal cells, which also could provide a source of Htt. Previous studies have shown that levels of mHtt in leukocyte blood fractions were significantly associated with disease burden scores and caudate atrophy rates in patients with HD^17^, hence our results could be consistent with these findings. However, similar findings in that study were not reported for levels of tHtt, nor were there any correlations found between mHtt levels in buccal cells and disease burden or striatal volume^17^, which would argue against buccal cells providing the majority of the measured tHtt. Nonetheless, it is important to note that one drawback of that study was the small sample size of manifest patients (n = 5)^17^. Mutant Htt levels have also been measured in plasma^14^. In that paper, the authors report no significant association between CSF and matched plasma mHtt concentrations, however their supplementary data indeed show a trend towards a correlation, with an r value = 0.454; p = 0.14^14^.

Additional sources of Htt could be the salivary glands themselves, which are also known to express Htt protein^6^, or, it is also possible that the nerves innervating salivary glands release Htt into the saliva. It is critical in future investigations to explore the precise sources and contributions of salivary Htt not only for understanding the fundamental mechanisms involved in the transportation of this protein, but also for controlling potential variables if salivary Htt could be used as a biomarker. The fact that tHtt levels in saliva in this study were found to be significantly associated with motor symptom scores, indicates its potential relevance to pathogenic and clinical events in the brain. However, we expect that salivary levels of the mutant only form might be more highly correlated with clinical data, and possibly even predictive of disease symptoms. This is especially important, when considering the need for a biomarker to monitor disease progression at the neuropathological level, and for future monitoring of possible disease modifying therapies.

Our results demonstrating increases in saliva levels of tHtt protein represent both the normal and mutant forms of the protein. Increases in tHtt could be due to several scenarios. Increased salivary tHtt protein could reflect increased expression of mutant or normal Htt protein, which has been reported previously in mouse models and brain tissue from HD subjects^27–29^. Alternatively, the elevated levels could be due to increased stability of mHtt in HD patients. Previous studies have demonstrated increased stability of mHtt in HD model systems and in human patients^30,31^. This increased stability of mHtt is thought to be due to abnormalities in the ubiquitin proteasomal system and autophagy systems, which are known to degrade the Htt protein, and such deficits have been reported in HD model systems^30–32^. Further, it is known that Htt is cleaved by numerous proteases^33,34^, and it is likely that smaller N-terminal Htt fragments are more stable than the full-length protein. It also must be considered that quantification of tHtt depends on, to some extent, on the protein size. Therefore, in absence of a set of standard recombinant tHtt proteins with different combinations of protein fragments, the calculated concentration of tHtt should be considered as relatively quantitative^35^. A consequence is that the most meaningful results will likely be obtained in situations where a single subject is monitored over time. Because we are measuring tHtt in soluble fractions of saliva, our data reflects the detection of soluble tHtt and would not represent aggregated forms of the protein, which could be present in the pellet fractions of saliva. Follow-up studies specifically measuring mutant forms and different cleavage products of Htt will be essential, in order to shed light on these issues.

We found significant correlations between salivary tHtt and age in our study. We believe this could be due to an accumulation of Htt with age, which could result from a reduced ability to clear Htt protein. Studies have reported that autophagy declines with aging^36,37^ which would be consistent with this notion. We also observed a trend towards a negative correlation between tHtt levels and CAG repeat length. We believe that this could be due to the positive correlation detected between tHtt levels and age in the HD patients, given that older patients typically have shorter CAG repeat lengths.

The possible effects of diet and medication status on salivary tHtt concentrations should also be considered. While we did not observe differences in salivary Htt in HD patients who were taking anti-depressant or anti-anxiety medications compared to drug-free patients (Fig. S2), the doses of these medications were not controlled for, hence detailed effects of medications remain to be determined. Some medications can reduce the output of saliva (i.e. reduced flow-rate), which might result in a more concentrated sample. However, we did not detect any correlations between saliva tHtt and total protein measurements in saliva, arguing against a reduced flow-rate contributing to the elevated levels observed in HD patients.

## Conclusion

In summary, measurements of salivary tHtt offer significant promise as a relevant, non-invasive disease biomarker for HD, and its use could be implemented into both clinical research and therapeutic applications. In particular, measurements of salivary tHtt could prove very valuable for upcoming clinical trials involving Htt-lowering strategies, whereby salivary readouts of tHtt or mHtt would be useful in assessing the effects of systemically delivered *HTT*-lowering molecules, or detecting peripheral effects of centrally delivered therapies.

## Methods

### Participants

This study was approved by the University of California, San Diego Institutional Review Board in accordance with the requirements of the Code of Federal Regulations on the Protection of Human Subjects. Informed consent from all subjects was obtained prior to their participation. Patients were recruited from the University of California, San Diego (UCSD) HD Clinical Research Center. HD patient criteria included a definitive diagnosis of HD with family history and an expanded trinucleotide CAG repeat of 40 or more. Normal controls had no reported history of neurological or psychiatric disorders, and no use of psychoactive substances or medications. Demographic information was collected at the time of saliva collection, including sex, age, medication status, CAG repeat length and age of onset. This information is provided in Table 1. There were no significant differences in the age or sex-ratios among the different diagnostic groups. Patients were assessed for cognitive and motor function using the Mini-Mental State Examination (MMSE; score range 0–30)^38^, the Unified Huntington’s Disease Rating Scale (UHDRS; score range 0–60),Total Functional Capacity (TFC; range 0–13) and overall disease burden using the Disease Burden Score (DBS).

### Sample collection

All donors were asked to refrain from smoking, eating, drinking, or oral hygiene procedures for at least 1 hour prior to samples collection, and then rinsed their mouth thoroughly with water 15–20 min prior to sample collection. Saliva samples were collected between 10:00 am and 4 pm. using the passive drool method according to previously established protocols^39^. Roughly two to three milliliters of unstimulated whole saliva was obtained. Samples were immediately frozen at −20 C at the time of collection, then stored at −80 C. At the time of use, saliva samples were thawed and centrifuged at 10,000 g for 10 min at 4 C to remove insoluble material and cellular debris. Saliva samples were only frozen a single time before use. Supernatants were collected and used for all assays. Total protein in the supernatants was determined used the BCA protein assay kit (Pierce).

### Western Blotting

Human saliva supernatants were concentrated 4-fold by vacuum centrifugation (4x) or used un-concentrated (1X, “neat”). For neat samples, 18 ul (~15 ug of protein) of saliva was loaded into each well and for concentrated samples, 18 ul (~60 ug of protein) were loaded per well. Samples were separated by 7% SDS-PAGE and blotted onto nitrocellulose membranes using standard procedures as described previously^32^. Ponceau’s stain was used to verify the transfer of proteins to the membrane. For immunodetection, the mouse anti-Htt antibody MAB2166 (Millipore;1:2,000 dilution) was used, followed by a goat anti-mouse HRP-conjugated secondary antibody (Pierce; 1:2,000 dilution). Immunoreactive bands were visualized using Pierce enhanced chemiluminescence (ECL) Western Blotting Substrate (Pierce). Gel images were acquired using a Fluorochem E Imager.

### ELISA measurements

Total Htt protein levels in saliva samples were quantified using a commercially available ELISA kit (LifeSpan BioSciences, Inc.) according to the manufacturer’s protocol using 50 ul of neat saliva per well diluted 1:1 with the provided Sample Dilution Buffer. The recombinant Htt protein standard corresponded to amino acids 802–940 of the human Htt protein, with antibodies corresponding to protein fragments including this region. The accuracy and precision of this ELISA in saliva was assessed by testing the recovery of a spiked-in control and the linearity of dilution of the spiked in control in two independent saliva samples. We found that the spiked-in recovery for the saliva matrix was 91.2% +/− 4.05% and the R^2^ value for linear regression = 0.976 (Fig. S1). Samples were assayed for cortisol using an immunoassay kit optimized for saliva (Salimetrics, LLC in Carlsbad, CA) following the manufacturer’s recommended protocol. All assays were performed by operators blinded to the clinical state of the participant.

### Statistics

All statistical analyses were performed using Graphpad software (Prism).Statistical significance was defined by a P value of less than 0.05. The lowest limit of detection in our assay was defined by two standard deviations above the zero value and was calculated to be 0.021 ng/ml. Any value below this number was excluded from further analysis. Excluded values included n = 3 control samples and n = 1 HD sample. The distribution of the data values in each diagnostic group was tested for normality using the Kolmogorov-Smirnov normality test. tHtt values in all groups, except the EPM group, showed a normal distribution. Differences between diagnostic groups were determined using One-way ANOVA followed by Dunnett’s post-test comparing all groups to the normal control group. Linear regression analysis (Spearman correlation) was used to compare tHtt protein levels against clinical variables, which were not normally distributed. Sex and medication differences were determined using Student’s *t* test (unpaired; two-tailed).

## Electronic supplementary material

Supplementary information
