# Supplementary material for: Salivary levels of total huntingtin are elevated in Huntington’s disease patients
Source: Sci Rep. 2018 May 9;8:7371. doi: 10.1038/s41598-018-25095-3 (PMC5943337; doi:10.1038/s41598-018-25095-3)
Supplement: Supplementary file 1 — Supplementary information [file 41598_2018_25095_MOESM1_ESM.pdf]

Title: Salivary levels of total huntingtin are elevated in Huntington's disease patients

Authors: Jody Corey-Bloom[1], Ameera Haque[1], Sungmee Park[1], Ajay Nathan[1], Robert Baker[2], Elizabeth A. Thomas[2]

Affiliations: [1] Department of Neurosciences, University of California, San Diego, CA,

[2]Department of Neuroscience, The Scripps Research Institute, La Jolla, CA.

*To whom correspondence should be addressed:
Elizabeth A. Thomas, Ph.D.
Department of Neuroscience
The Scripps Research Institute
10550 N. Torrey Pines Rd., SP2030
La Jolla, CA.
bthomas@scripps.edu

Figure S1. Western blot of Htt protein in saliva from normal and HD individuals. Saliva supernatants were used as-is (1x) or were concentrated 4-fold by vacuum centrifugation from 4 normal subjects (C5-C8) in (A), and in matched HD and control pairs (n=3) in (B). Supernatant fractions were separated by 7% SDS-PAGE and blotted onto nitrocellulose membranes for immunodetection using MAB2166 antibody (1:1,000) (A) or the 4E10 antibody (1:1,000) and beta-actin (1:1,000) (B). Gel image was acquired using a Fluorochem E imager.

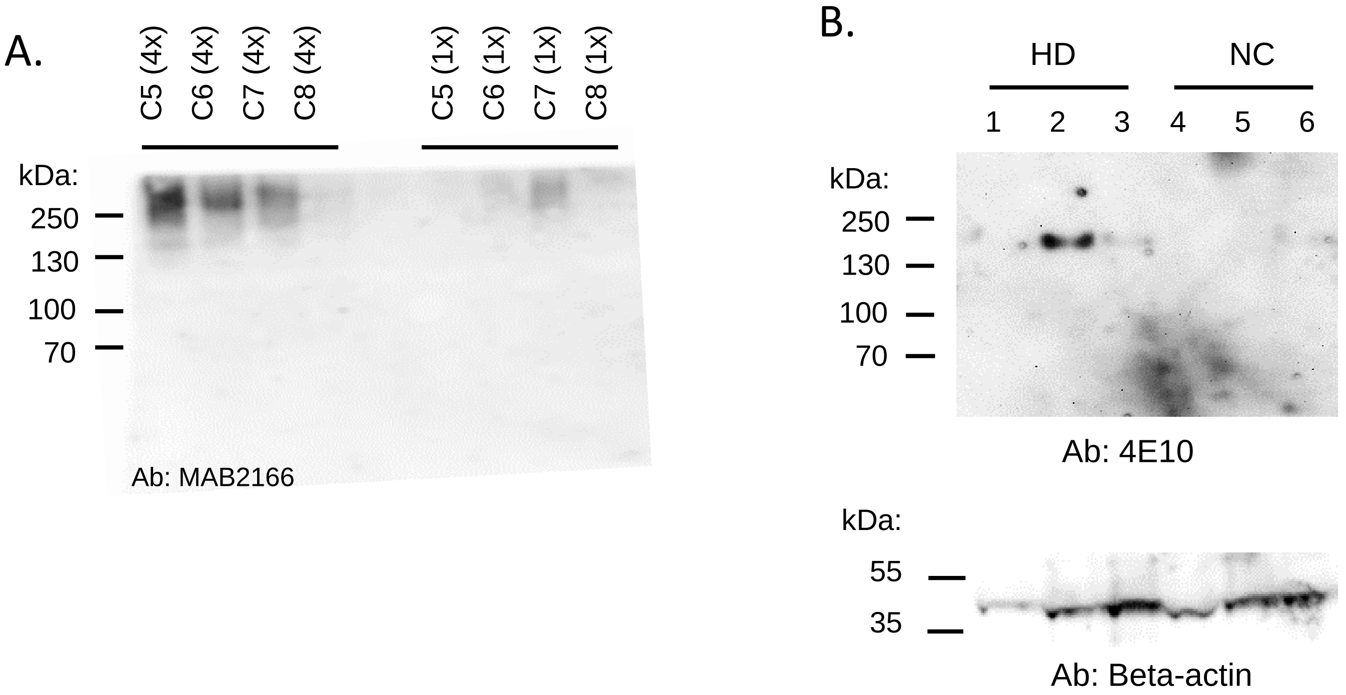

Figure S2. Levels of tHtt in medicated versus unmedicated HD patients. Levels of tHtt were determined by ELISA. The amount of tHtt (ng/ml) reported is relative to a recombinant Htt protein of 139 amino acids per the ELISA assay. We did not observe differences in salivary tHtt in HD patients who were taking anti-depressant or anti-anxiety medications compared to drug-free patients.

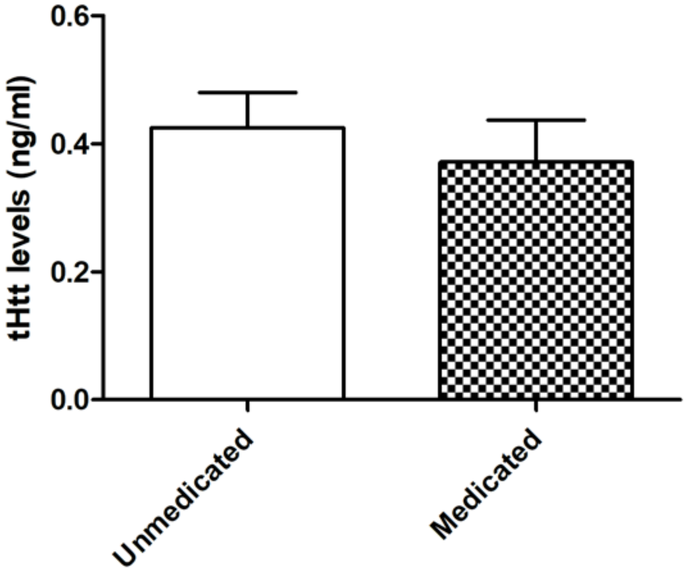

Figure S3. Linearity of dilution of tHtt in saliva. Levels of tHtt were determined by ELISA. The amount of tHtt (ng/ml) reported is relative to a recombinant Htt protein of 139 amino acids per the ELISA assay. The spiked-in recovery for the saliva matrix was 91.2% +/- 4.05% and the R^2 value for linear regression = 0.976.

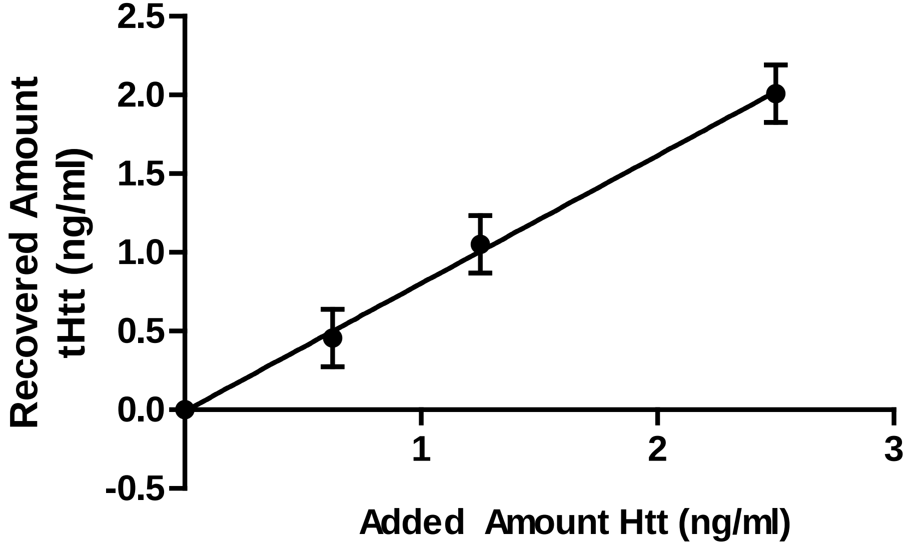

Table S1. Clinical data from the subjects used in this study.

		COHORT 1										
		**CAG repeat #**	**Age of Onset (yrs)**	**PAO (yrs)**	**Langbehn # (yrs)**	**CAP**	**DBS**	**TFC**	**MMSE**	**Motor**	**DCL**	**tHtt (ng/ml)**
**HD**	Mean	43.167	48.208	48.696	6.575	484.667	385.000	9.250	26.667	32.083	4.000	0.547
**N=23**	SD	3.031	11.538	14.294	3.772	85.144	92.240	2.005	2.808	13.227	0.000	0.079
	Min	37.000	22	30	3.930	220.440	99.000	6	20	9	4	0.043
	Max	50.000	68	70	23.000	678.700	577.500	13	30	63	4	1.530
**LPM**	Mean	42.250	53.625	51.375	7.183	452.285	350.625	12.000	28.250	10.375	3.000	0.425
**N=9**	SD	2.315	14.667	14.071	1.357	30.338	41.552	1.195	1.832	3.926	0.926	0.112
	Min	40.000	36	38	5.14	396.36	297.00	10	25	6	2	0.048
	Max	47.000	71	70	8.98	485.68	414.00	13	30	17	4	1.240
**EPM**	Mean	41.222		55.111	18.548	303.876	225.778	12.889	28.111	2.222	0.444	0.436
**N=10**	SD	3.420		13.941	12.261	116.381	113.274	0.333	1.764	1.716	0.527	0.133
	Min	38.000		32	4.97	165.54	105.00	12	25	0	0	0.149
	Max	48.000		75	37.86	487.56	425.00	13	30	5	1	1.520
**AR-**	Mean	23.077						13.000	28.813	1.563	0.250	0.368
**N=16**	SD	6.157						0.000	1.601	1.153	0.447	0.073
	Min	17.000						13	25	0	0	0.021
	Max	33.000						13	30	4	1	0.935
**NC**	Mean							12.667	28.611	2.444	0.278	0.340
**N=30**	SD							0.767	2.570	2.684	0.461	0.046
	Min							10	20	0	0	0.028
	Max							13	30	9	1	1.070

Table S1, cont. Clinical data from the subjects used in this study.

	COHORT 2											
		CAG repeat #	Age of Onset (yrs)	PAO (yrs)	Langbehn # (yrs)	CAP	DBS	TFC	MMSE	Motor	DCL	tHtt (ng/ml)
**HD**	Mean	42.250	52.750	58.875	6.231	485.938	376.688	8.500	24.429	33.500	4.000	0.593
**N=14**	SD	2.816	11.732	13.632	1.268	46.951	58.990	3.505	2.507	11.314	0.000	0.077
	Min	40.000	28	27	3.940	411.040	308.000	2	19	15	4	0.293
	Max	49.000	68	70	8.220	567.580	499.500	13	27	52	4	1.320
**LPM**	Mean	42.000	38.000	45.000	12.810	341.940	266.500	13.000	27.000	14.000	4.000	0.732
**N=1**	SD											
	Min											
	Max											
**EPM**	Mean	41.400		52.667	20.085	323.995	245.733	13.000	28.333	1.933	0.467	0.462
**N=16**	SD	1.724		12.838	15.452	83.306	75.765	0.000	1.496	1.831	0.640	0.055
	Min	38.000		35	7.16	165.54	108.50	13	25	0	0	0.106
	Max	44.000		75	66.47	448.32	360.00	13	30	6	2	0.881
**AR-**	Mean	19.800						13.000	28.400	0.800	0.200	
**N=13**	SD	3.834						0.000	1.517	1.304	0.447	
	Min	17.000						13	26	0	0	
	Max	26.000						13	30	3	1	
**NC**	Mean							12.909	28.727	1.909	0.182	0.378
**N=14**	SD							0.302	0.905	1.814	0.405	0.068
	Min							12	27	0	0	0.029
	Max							13	30	5	1	0.729

HD, Huntington's disease; LPM, late pre-manifest; EPM, early pre-manifest; AR-, at-risk, gene negative; NC, normal controls.
PAO, paternal age of onset; CAP, normalized CAG Age Product; DBS, disease burden score; TFC, total functional capacity score.
MMSE, mini-mental state exam; Motor, motor symptom score; DCL, diagnostic confidence level.
"Langbehn #" is based on a formula that estimates years to onset in HD (Langbehn DR et al., Clin Genet. 65:267-77, 2004).
